# Chemical Recycling of Post-Consumer Polystyrene by Thermal Pyrolysis: High-Yield Recovery of Aromatic Hydrocarbons for Circular Plastic Economy

**DOI:** 10.3390/polym18101172

**Published:** 2026-05-09

**Authors:** Joaquin Hernandez-Fernandez, Rafael Gonzalez-Cuello, Rodrigo Ortega-Toro

**Affiliations:** 1 Chemistry Program, Department of Natural and Exact Sciences, University of Cartagena, San Pablo Campus, Cartagena de Indias 130015, Colombia; 2 Department of Natural and Exact Science, Universidad de la Costa, Barranquilla 080002, Colombia; 3 Food Packaging and Shelf-Life Research Group (FP & SL), Food Engineering Program, University of Cartagena, Cartagena de Indias 130015, Colombia; rgonzalezc1@unicartagena.edu.co (R.G.-C.); rortegap1@unicartagena.edu.co (R.O.-T.)

**Keywords:** post-consumer polystyrene, thermal pyrolysis, chemical recycling, fixed-bed reactor, aromatic hydrocarbons, styrene recovery, waste valorization, circular economy

## Abstract

This study evaluates the non-catalytic thermal pyrolysis of post-consumer polystyrene (PS) in a laboratory-scale batch fixed-bed reactor to recover aromatic-rich liquid products. The PS feedstock was characterized by thermogravimetric analysis (TGA) and micro-Raman spectroscopy to assess its thermal behavior and chemical homogeneity. In addition, the main TGA degradation region was analyzed using Coats–Redfern, Horowitz–Metzger, and Broido kinetic models, yielding apparent activation energies of 269.18, 288.83, and 280.69 kJ mol^−1^, respectively. Pyrolysis experiments were performed at final temperatures of 400, 450, and 500 °C and heating rates of 10 and 20 °C min^−1^ under continuous N_2_ flow. The maximum liquid yield reached 95.2 wt% at 500 °C and 20 °C min^−1^, while the estimated gaseous fraction decreased to approximately 2.0 wt%. ANOVA confirmed that final temperature was the dominant factor controlling liquid recovery, contributing approximately 83% of the model variability, whereas heating rate had a secondary but significant effect. GC–MS analysis showed that the pyrolysis oil was mainly composed of aromatic hydrocarbons, including styrene, toluene, and ethylbenzene, with increasing temperature promoting the redistribution of the liquid fraction toward lighter monoaromatic compounds. These results indicate that non-catalytic fixed-bed pyrolysis is a promising route for converting post-consumer PS into aromatic-rich liquid products. However, the recovered oil should be considered a complex mixture rather than a purified monomer stream, and further gas-phase characterization, downstream purification, energy-balance evaluation, life-cycle assessment, and techno-economic analysis are required before definitive claims regarding industrial circularity or environmental performance can be established.

## 1. Introduction

The global proliferation of plastic waste has become one of the most critical environmental challenges of the 21st century. In this context, polystyrene (PS) constitutes an important fraction due to its extensive use in packaging, electronics, insulation, and industrial applications [1]. Although mechanical recycling has traditionally been the most common route for plastic waste management, its effectiveness is limited by the progressive degradation of polymer properties during reprocessing, the complexity of sorting and cleaning post-consumer streams, and the generally low value of mechanically recycled products [2,3]. These limitations have increased interest in chemical recycling technologies that can convert PS waste into higher-value aromatic compounds and liquid fractions [4,5].

Thermal pyrolysis is one of the most studied chemical recycling routes for PS because this polymer predominantly decomposes via depolymerization reactions, producing styrene as the primary product, along with other aromatic hydrocarbons such as toluene, ethylbenzene, α-methylstyrene, and styrene-derived oligomers [6,7]. Previous studies have shown that PS begins to degrade in the 350–400 °C range and can yield liquids above 90 wt.% under optimized conditions [8,9,10]. However, the distribution of pyrolysis products is highly sensitive to operating conditions, including final temperature, heating rate, vapor residence time, pressure, carrier gas flow, and condensation efficiency. In particular, styrene selectivity can vary considerably because primary depolymerization reactions compete with secondary radical pathways, hydrogen-transfer reactions, oligomer cracking, and overcracking processes [6,8].

Although catalytic and thermo-catalytic pyrolysis have been widely investigated to improve selectivity and reduce the apparent thermal severity of PS conversion, catalytic systems may also involve additional operational challenges, including catalyst cost, deactivation, regeneration requirements, and sensitivity to feedstock impurities [7,11]. Therefore, non-catalytic fixed-bed pyrolysis remains relevant as a simpler reference process for evaluating the intrinsic thermal conversion behavior of post-consumer PS and for identifying operating conditions that favor liquid recovery before introducing additional catalytic complexity.

The novelty of this study does not rely on the isolated use of gas chromatography–mass spectrometry or on evaluating temperature and heating rate alone, since these approaches are already established in PS pyrolysis research. Instead, this work contributes by integrating the evaluation of product mass balance, GC–MS-based aromatic fraction analysis, thermogravimetric and micro-Raman characterization of the post-consumer PS feedstock, and statistical interpretation using correlation analysis and ANOVA. This combined approach allows the effect of final temperature and heating rate on liquid yield and aromatic product distribution to be assessed in a fixed-bed batch reactor under controlled non-catalytic conditions. The experiments were conducted at final temperatures of 400, 450, and 500 °C and heating rates of 10 and 20 °C min^−1^ under a constant N_2_ atmosphere. This experimental design enabled the identification of a thermal operating window that favors high liquid recovery and provides information on the redistribution of the aromatic fraction from heavier oligomeric compounds toward lighter monoaromatic products.

Therefore, this study aims to evaluate the effect of final temperature and heating rate on the thermal pyrolysis of post-consumer PS in a fixed-bed reactor, with emphasis on liquid yield, aromatic hydrocarbon distribution, and the statistical significance of the operating variables. The results are discussed as a basis for assessing the potential of PS pyrolysis oil as a source of aromatic chemical feedstocks or fuel-range components. However, because no life-cycle assessment, full energy balance, or economic feasibility analysis was performed, the environmental and industrial implications of the process are presented as potential advantages rather than as demonstrated outcomes [12,13,14,15].

## 2. Materials and Methods

### 2.1. Raw Materials and Preparation

The polymer precursor material comprised post-consumer polystyrene obtained from industrial packaging waste [1]. The collected material mainly consisted of protective packaging residues and did not include food-contact waste or samples visibly contaminated with organic residues, such as food remains, oils, or grease. Before pyrolysis, the PS waste was manually inspected to remove labels, foreign particles, and non-polymeric impurities. The selected material was then washed with distilled water and dried at 60 °C for 24 h to remove residual moisture and superficial contaminants. Finally, the material was mechanically ground to a particle size of approximately 2–5 mm to ensure uniform heat transfer during thermal degradation [11,16].

#### 2.1.1. TGA

Thermogravimetric analysis (TGA) was performed using a Discovery 55 TGA thermobalance (TA Instruments, New Castle, DE, USA), over a temperature range of 25 to 900 °C at a controlled heating rate of 20 °C min−1. Instrument control and data processing were carried out using TRIOS software (version 5.10.0.0450, TA Instruments, New Castle, DE, USA). Nitrogen (N_2_; 99.95–99.9990%, Messer Colombia S.A., Bogotá, Colombia) was used as the carrier gas at a precisely regulated flow rate of 0.83 ± 0.005 mL s^−1^. This analysis provided information on the thermal stability and degradation behavior of the analyzed material.

#### 2.1.2. Kinetic Analysis of TGA Data

The thermal degradation kinetics of post-consumer polystyrene were evaluated from the non-isothermal TGA curve obtained under nitrogen atmosphere. Three integral kinetic models were applied to the main degradation region: Coats–Redfern, Horowitz–Metzger, and Broido. These models were selected to provide a comparative estimation of the apparent activation energy associated with the dominant thermal decomposition event of the PS sample [10,17,18].

The conversion degree, α, was calculated from the normalized mass loss according to:

α = (*W*_0_ − *W*_t_)/(*W*_0_ − *W*f)

where *W*_0_ is the initial sample mass, *W*_t_ is the sample mass at temperature *T*, and *W*f is the final residual mass after thermal degradation. The kinetic treatment was based on the general solid-state degradation equation:

dα/d*t* = *A* exp(−*E*a/*RT*) *f*(α)

where *A* is the pre-exponential factor, *E*a is the apparent activation energy, *R* is the universal gas constant, *T* is the absolute temperature, and *f*(α) is the reaction model. Under a constant heating rate, *β* = d*T*/d*t*, this expression can be rewritten as:

dα/d*T* = (*A*/*β*) exp(−*E*a/*RT*) *f*(α)

For each kinetic model, the corresponding linearized equation was applied to the main mass-loss region of the TGA curve, as summarized in Table 1. The apparent activation energy was obtained from the slope of the linear regression, while the goodness of fit was evaluated using the coefficient of determination, R^2^. Because the kinetic analysis was performed using a single heating rate, the calculated activation energies were interpreted as apparent values associated with the dominant degradation step rather than as model-independent kinetic parameters [10,17,19,20].

#### 2.1.3. Micro-Raman

Raman measurements were performed using a DXRT™ Raman Microscope (Thermo Fisher Scientific, Waltham, MA, USA) with 532 nm excitation (green laser). The laser radiation was focused using 50× and 100× objectives (numerical apertures (NAs) of 0.50 and 0.90, respectively; NA defines how much light the lens can gather). Laser power and acquisition settings were optimized through preliminary tests within the reported ranges.

### 2.2. Experimental System and Reactor

**Figure 1:**
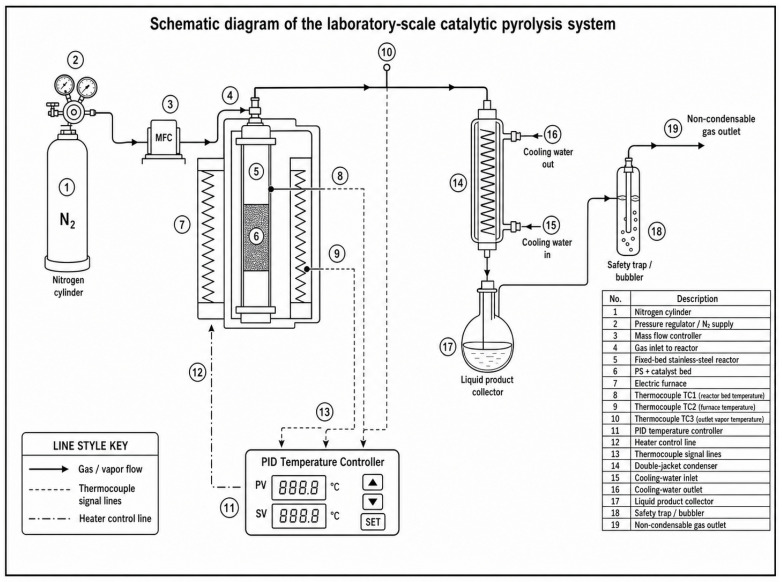
Schematic representation of the laboratory-scale fixed-bed pyrolysis system used for post-consumer PS conversion. The numbered elements correspond to the N_2_ cylinder, pressure regulator, mass flow controller, reactor gas inlet, fixed-bed stainless-steel reactor, PS/catalyst bed, electric furnace, thermocouples TC1–TC3, PID temperature controller, double-jacket condenser, liquid-product collector, safety trap/bubbler, and non-condensable gas outlet. The N_2_ stream was introduced directly into the reactor inlet, while TC1, TC2, and TC3 monitored the reactor-bed temperature, furnace temperature, and outlet-vapor temperature, respectively. Thermocouple signals were connected to the PID controller for temperature monitoring and furnace regulation.

The pyrolysis experiments were conducted in a laboratory-scale batch fixed-bed reactor designed for the thermal conversion of post-consumer PS in an inert atmosphere. Based on laboratory-scale fixed-bed/batch systems previously described for PS pyrolysis, the reactor was designed as a high-temperature-resistant stainless steel cylindrical vessel with an internal height of approximately 150 mm, an internal diameter of approximately 63.5 mm, and a maximum loading capacity of approximately 150 g of polymeric material [2,21]. In each experiment, 50 g of prepared PS was loaded into the reactor, corresponding to approximately 33% of the reactor’s estimated maximum capacity. This partial loading was chosen to prevent excessive bed compaction, facilitate vapor release, and reduce potential blockage of the vapor outlet during thermal degradation. The reactor was externally heated using an electric furnace equipped with a PID temperature controller, enabling programmed heating ramps of 10 and 20 °C min−1 to reach the selected final temperatures of 400, 450, and 500 °C. Similar fixed-bed and semi-batch PS pyrolysis studies have reported the use of electrically heated stainless-steel reactors, thermocouple monitoring, and PID-based temperature control to ensure reproducible thermal conditions during polymer degradation [21,22,23]. After reaching the target temperature, the system was held isothermally for 30 min to promote extensive thermal degradation under comparable operating conditions. This holding time is consistent with fixed-bed PS pyrolysis studies, in which residence times of approximately 30 min have been used to evaluate product yields and oil composition. The condensation system consisted of a double-jacketed glass condenser connected to a circulating cooling bath maintained at 5 °C, consistent with laboratory-scale PS pyrolysis configurations that use rapid cooling to recover condensable vapors as pyrolysis oil. For reference, previously reported PS pyrolysis systems have used condenser geometries of approximately 305 mm in height and 63.5 mm in diameter [24]. In the present setup, the condensed liquid fraction was collected in a receiving vessel, while non-condensable gases were directed toward a safety trap before release. The liquid receiver and reactor residue were weighed after cooling to determine the liquid and solid fractions, respectively. In contrast, the gaseous fraction was estimated by mass balance, as described in Section 2.4. A schematic representation of the laboratory-scale fixed-bed pyrolysis system is shown in Figure 1.

### 2.3. Experimental Design and Operating Conditions

The effects of reaction temperature and heating rate for a factorial experimental design were established. For the final pyrolysis, temperatures were set to 400 °C, 450 °C, and 500 °C, with heating rates of 10 °C min^−1^ and 20 °C min^−1^. After reaching the target temperature, the system was held isothermally for 30 min to ensure complete conversion of the raw material [25,26]. The condensation system was kept at 5 °C in a circulating coolant bath to maximize recovery of the liquid fraction (pyrolysis oil) and minimize losses of volatile compounds [27,28].

The experimental matrix was designed to evaluate the specific effects of final temperature and heating rate on the product yield distribution of post-consumer PS pyrolysis. Other operational parameters, including particle size, N_2_ flow rate, reactor loading, condensation temperature, and holding time, were kept constant to reduce experimental variability and allow direct comparison between the selected thermal conditions. The particle size range of 2–5 mm was selected to improve packing homogeneity and heat transfer while avoiding excessive fines that could obstruct the fixed bed or increase uncontrolled pressure drop [11,16]. The N_2_ flow rate was fixed at 100 mL min^−1^ to maintain an inert atmosphere and assist the removal of pyrolysis vapors from the hot zone. The system was held isothermally for 30 min after reaching the target temperature to promote extensive thermal degradation under comparable operating conditions [29,30,31]. However, vapor residence time was not independently varied or directly quantified in this study, and the selected N_2_ flow rate and holding time should be interpreted as fixed operating conditions rather than optimized parameters.

### 2.4. Product Quantification and Characterization: GC-MS Analysis of Oil

The yields of the products (liquid, solid, and gas) were determined using mass balance, following the methodology. Liquid yield (YL): weight of the oil collected in the condensers. Solid yield (YS): calculated as the weight of the carbonaceous residue (char) remaining in the reactor. Gas yield (YG): calculated by difference (YG = 100 − YL − YS).

The product yields were determined by mass balance. Liquid yield (YL) was calculated from the mass of pyrolysis oil collected in the condensation system. In contrast, solid yield (YS) was determined from the mass of carbonaceous residue remaining in the reactor after cooling. The gaseous yield (YG) was estimated by difference according to YG = 100 − YL − YS. Since the gaseous fraction was not collected or directly analyzed, YG was considered an estimated gaseous yield calculated from the liquid and solid fractions. Therefore, this value may include cumulative experimental deviations associated with liquid collection, residual material recovery, condensation efficiency, and minor volatile losses during product transfer. To minimize these deviations, the condensation system was maintained at 5 °C, the receiver and reactor residue were weighed after cooling, and the same collection and weighing procedure was applied to all experiments. Accordingly, the gaseous fraction is discussed as an estimated mass-balance term rather than as a directly quantified gas stream.

The chemical composition of the pyrolytic oil was analyzed by gas chromatography–mass spectrometry using an Agilent 7890B system (Santa Clara, CA, USA). A DB-5MS capillary column (30 m × 0.25 mm × 0.25 µm) was used with helium as the carrier gas. The oven temperature program was configured to start at 40 °C, hold for 2 min, then increase to 300 °C at 10 °C min^−1^ [6,8]. The identification of the main aromatic compounds, including styrene, toluene, and ethylbenzene, was performed by comparing their mass spectra with the NIST library database. Special attention was paid to identifying and estimating the relative abundance of styrene in the pyrolysis oil, since styrene is one of the main target compounds in PS chemical recycling [8,11].

## 3. Results and Discussion

### 3.1. Sampling and Characterization of Post-Consumer PS: TGA and Micro-Raman

**Figure 2:**
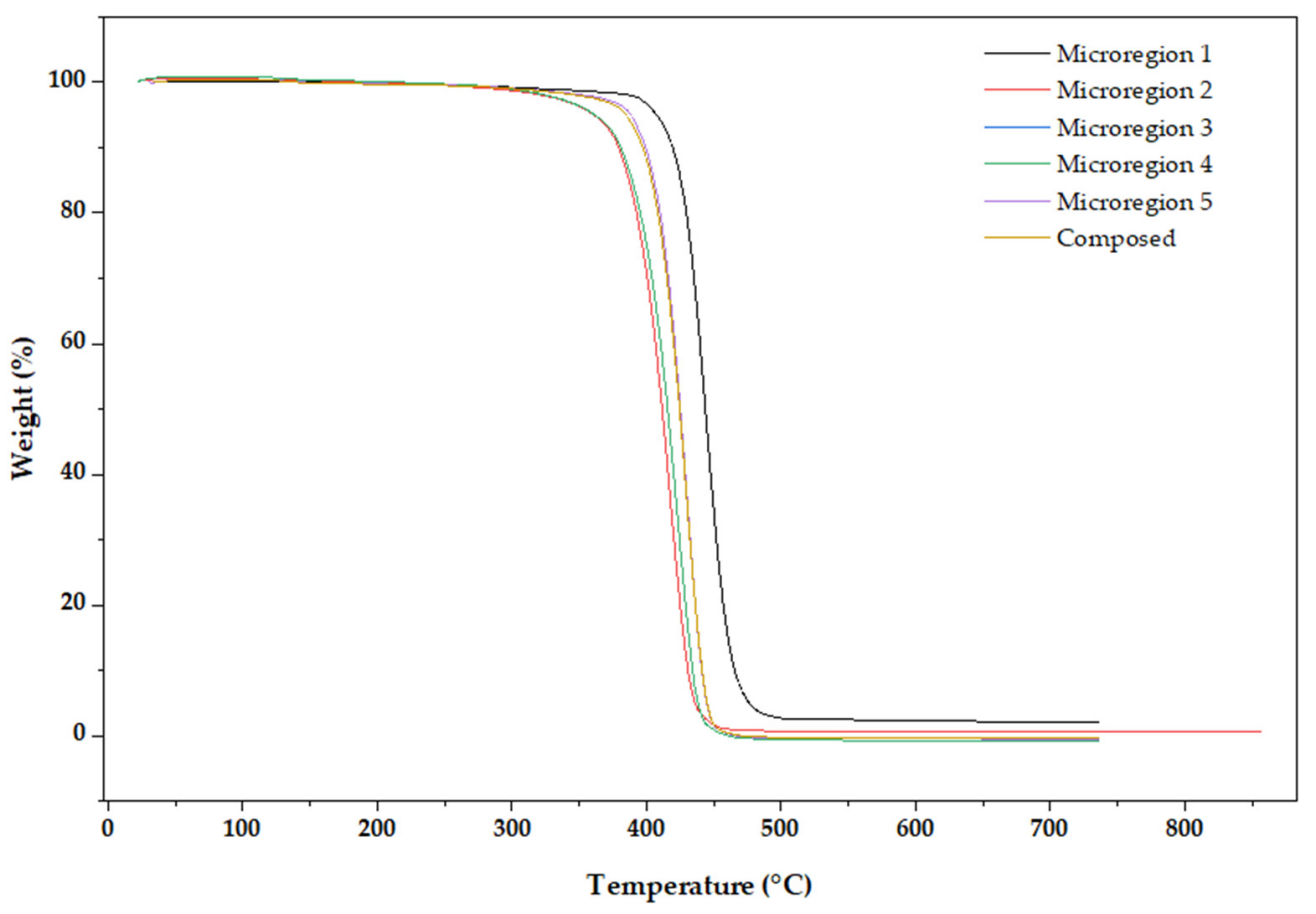
Thermogravimetric curves (TGA) correspond to Microregions 1–5 and the average curve obtained from all the sampling areas of the studied material.

Figure 2 shows that the analyzed microregions exhibited a similar thermal degradation profile, characterized by a single main mass-loss event after an initial region of thermal stability. This behavior is consistent with the dominant decomposition of PS through chain scission and volatilization processes [15,24]. The relevance of the TGA results for the pyrolysis experiments lies in two main aspects. First, the onset and main degradation region support selecting the 400–500 °C range for the fixed-bed reactor, as this interval encompasses the temperature range over which PS undergoes extensive thermal decomposition. Second, the low final residue observed in the thermogravimetric curves indicates a low tendency toward char formation, which is consistent with the low solid yields obtained during pyrolysis. Therefore, TGA was used not only as a characterization tool but also as a preliminary thermal criterion to justify the operating temperature window and to interpret the limited formation of solid residue during the process [29,30,31,32,33].

**Figure 3:**
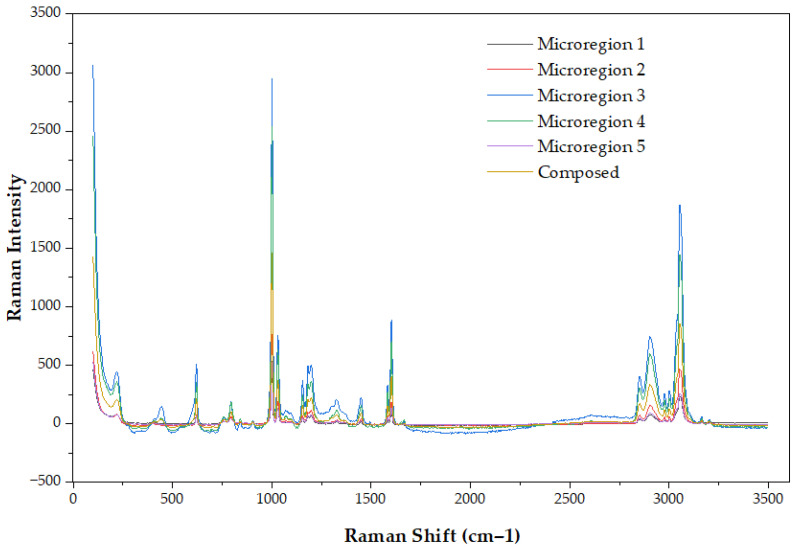
Micro-Raman spectra corresponding to Microregions 1–5 and the average spectrum obtained from all the sampling areas of the studied material, recorded in the range of 100–3500 cm^−1^.

Figure 3 shows that the Raman spectra collected from the different microregions presented the characteristic vibrational fingerprint of PS, including bands associated with aromatic ring vibrations and C–H stretching modes. The absence of new bands or significant spectral shifts among the analyzed microregions indicates that the feedstock was chemically homogeneous at the analyzed scale. At the same time, the observed intensity differences can be attributed mainly to local physical effects such as surface roughness, thickness, orientation, or focusing conditions [34,35,36,37,38,39,40,41]. This information is relevant for the pyrolysis interpretation because it confirms that the observed differences in product yield and liquid composition are more reasonably associated with the selected operating variables, particularly final temperature and heating rate, rather than with major chemical heterogeneity of the starting material. Thus, Raman spectroscopy supports the validity of comparing the pyrolysis runs under a common chemical feedstock basis.

Taken together, TGA and micro-Raman analyses provided the feedstock-level evidence required to interpret the pyrolysis results. TGA supported the selection of the thermal operating range and the interpretation of low solid residue formation. In contrast, micro-Raman confirmed the chemical identity and relative homogeneity of the post-consumer PS feedstock. Accordingly, these techniques were used as supporting tools for process interpretation rather than as direct measurements of pyrolysis yield.

To provide kinetic support for the thermal behavior observed by TGA, the main degradation region of post-consumer PS was analyzed using three integral kinetic models: Coats–Redfern, Horowitz–Metzger, and Broido. The calculated values were interpreted as apparent activation energies because the analysis was performed from non-isothermal TGA data obtained at a single heating rate. Therefore, the kinetic parameters should not be considered model-independent values, but rather comparative indicators of the energetic demand associated with the dominant thermal degradation event of the PS sample [19,20]. The apparent activation energies obtained from the three kinetic models are summarized in Table 2.

The apparent activation energies obtained from the three models, summarized in Table 2, were 269.18–288.83 kJ mol^−1^, indicating a consistent kinetic response for the main degradation event. The Coats–Redfern model produced the lowest Ea value, 269.18 kJ mol^−1^, whereas the Horowitz–Metzger model gave the highest value, 288.83 kJ mol^−1^. The Broido model yielded an intermediate value of 280.69 kJ mol^−1^. The relatively narrow difference among the three models suggests that the analyzed mass-loss event corresponds to a dominant degradation process rather than to multiple highly divergent thermal events [17].

From a chemical perspective, these activation energies are consistent with the thermal cleavage of the PS backbone and subsequent volatilization of aromatic degradation products. The relatively high Ea values indicate that substantial thermal energy is required to initiate and propagate the degradation process, which supports the selection of the 400–500 °C range for the fixed-bed pyrolysis experiments. In this sense, the kinetic analysis complements the TGA profile by providing quantitative evidence that the selected pyrolysis window covers the main energetic region associated with PS decomposition [9,29].

The kinetic analysis of the individual microregions, summarized in Table 3, revealed moderate spatial variability in the apparent activation energy. For all three models, Microregion 1 showed the highest Ea values, reaching 297.23 kJ mol^−1^ by Coats–Redfern, 311.83 kJ mol^−1^ by Horowitz–Metzger, and 309.11 kJ mol^−1^ by Broido. In contrast, Microregion 4 presented the lowest values, with 207.34, 228.91, and 218.66 kJ mol^−1^, respectively. This trend indicates that some local areas of the sample required slightly higher thermal energy for degradation, whereas others degraded more readily [17].

Despite these local differences, the same relative order was observed across the three models, which supports the internal consistency of the kinetic analysis. The general trend can be summarized as follows: Microregion 1 > Microregion 5 > Microregion 3 ≈ Composite curve > Microregion 2 > Microregion 4. This agreement indicates that the differences among microregions are not random artifacts of a single kinetic model, but reflect measurable local variations in thermal response [17,19].

The composite curve produced intermediate activation energy values, matching those of Microregion 3 in this dataset. This behavior suggests that the composite TGA response adequately represents the material’s average degradation behavior. Therefore, although the post-consumer PS sample exhibited some local variability, the global kinetic response remained coherent with a dominant PS degradation process. This interpretation is also consistent with the micro-Raman results, which confirmed the characteristic vibrational fingerprint of PS across the analyzed microregions. Thus, the local differences in Ea are more reasonably attributed to physical or microstructural effects, such as local packing, thickness, surface morphology, or heat-transfer differences, rather than to major chemical heterogeneity of the feedstock.

Overall, the kinetic results strengthen the relevance of the TGA for interpreting the pyrolysis experiments. The activation energy values confirm that the main degradation of post-consumer PS requires a high thermal input. At the same time, the consistency among the three models supports the reliability of the selected degradation region. These results justify the use of the 400–500 °C experimental range and provide a kinetic basis for understanding why increasing the final pyrolysis temperature favored liquid recovery in the fixed-bed reactor.

### 3.2. Effect of Temperature and Heating Rate on Thermal Pyrolysis Yields

**Figure 4:**
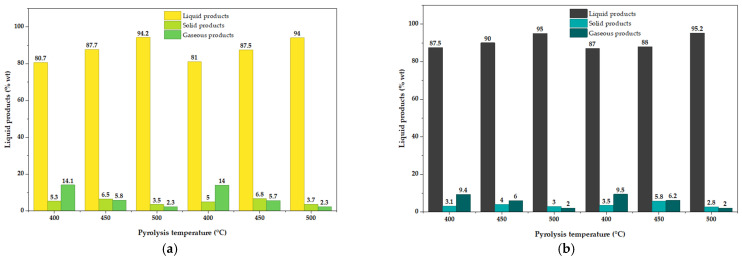
Product yield distribution from PS thermal pyrolysis at different heating rates: (**a**) 10 °C min^−1^ and (**b**) 20 °C min^−1^. Bar colors identify liquid, solid, and estimated gaseous products.

The product yield distribution obtained under each experimental condition is summarized in Table 4 and graphically represented in Figure 4. This organization allows the direct comparison of liquid, solid, and estimated gaseous fractions as a function of final temperature and heating rate. Overall, increasing the final temperature from 400 to 500 °C favored the recovery of the liquid fraction, with the maximum liquid yield reaching 95.2 wt.% at 500 °C and 20 °C min^−1^ [21,38,39,40,41,42,43,44]. These results are higher than the literature averages reported for conventional batch pyrolysis systems; for example, PS oil yields under comparable conditions have been reported to range from 82 to 92 wt.% [15]. Compared with studies employing standard fixed-bed reactors at 500 °C, the liquid recovery obtained in this work represents an improvement of approximately 3.2–15.8% [6].

Hence, these results show that heating rate influenced liquid yield mainly at lower final temperatures. His project presents the impact of heating rate on conversion efficiency at low temperatures. The liquid yield increased from 80.8% to 87.2% at 400 °C (an increase of 6.4% in absolute value, from 10 to 20 °C min^−1^). It is far superior to the results of previous studies on thermal kinetics, which show that slower rates tend to promote secondary reactions and char formation. As in our case, we find that the 20 °C min^−1^ rate minimizes the residence time of vapors in the high-temperature zone, reduces secondary cracking into gases, and enables the best possible aromatic condensation [6]. This is an operational benefit because it enables extremely high yields (87.2%) at lower temperatures (400 °C), reducing energy demand in the industrial process.

The estimated gaseous fraction decreased to approximately 2.0 wt% at 500 °C, suggesting that the evaluated conditions favored condensable liquid formation over non-condensable products. However, this value should be interpreted cautiously because the gas phase was not directly collected, quantified, or compositionally analyzed in this study. Instead, the gaseous fraction was calculated by difference from the liquid and solid yields, and therefore represents an estimated mass-balance term that may include cumulative deviations associated with liquid condensation, product recovery, and minor volatile losses. Consequently, direct gas collection and compositional analysis would be required to validate the absolute gas yield and determine the specific gas-phase composition.

The recovery of an aromatic-rich liquid fraction suggests that the pyrolysis oil could serve as a potential source of chemical feedstocks, particularly after appropriate downstream separation and purification steps. Although styrene recovery is relevant for plastic-to-plastic recycling concepts, the liquid product obtained in this study is a complex mixture rather than a purified monomer stream. Therefore, the present results support the potential valorization of PS-derived pyrolysis oil, but they do not by themselves demonstrate direct industrial monomer recovery or closed-loop circularity.

**Figure 5:**
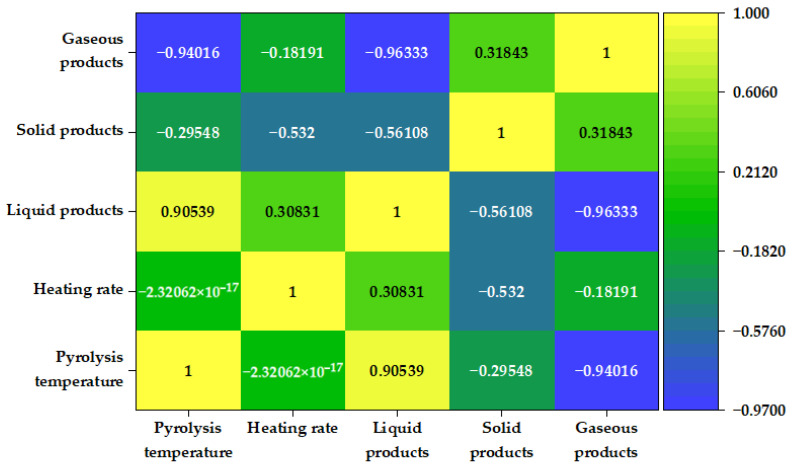
Correlation heatmaps for PS pyrolysis products.

Figure 5 presents a correlation matrix summarizing the relationships between the operating variables and the product distribution during PS pyrolysis. The most evident trend is a clear positive correlation between pyrolysis temperature and liquid yield (r = 0.905), confirming that temperature was the most significant parameter influencing liquid yield in the 400–500 °C range. This result is consistent with the known thermal behavior of polystyrene, wherein chain scission and depolymerization reactions produce more condensable aromatic compounds, particularly near 500 °C [15,45]. The maximum liquid yields, nearly 95.2 wt%, were obtained at 500 °C, suggesting the influence of temperature on the process variable [6,8]. The temperature also exhibited a strong negative correlation with gaseous products (r = −0.940), indicating that, within the present experimental window, increasing the temperature shifted the product distribution toward condensable liquids rather than toward estimated gaseous products. This trend is especially relevant because it shows that the system operated below the regime in which excessive secondary cracking would become dominant. Instead of shifting the reaction network toward gas overproduction, the increase from 400 to 500 °C reduced the gas fraction from about 14 wt.% to nearly 2 wt.%, while simultaneously increasing the liquid fraction. This response is characteristic of a favorable thermal depolymerization window for PS, in which the supplied energy enhances volatilization and the formation of condensable aromatic products without intensifying overcracking into non-condensable species [6,8].

A very strong inverse correlation was also found between liquid and gaseous products (r = −0.963). This result confirms that the conditions favoring oil production are associated with a marked suppression of gas formation. However, this relationship should be interpreted with caution, since the gaseous fraction was calculated by difference from the liquid and solid fractions, which inherently introduces compositional dependence among variables. Therefore, although the magnitude of this coefficient is useful for visualizing the mass-balance trend of the process, it should not be considered independent mechanistic proof by itself. Even so, from a process standpoint, the trend clearly indicates that operational conditions that lead to higher liquid recovery also minimize carbon losses to the least-valuable stream [6,44]. The influence of heating rate on liquid yield was relatively weaker (r = 0.308), suggesting that it acts secondarily to final temperature. While not irrelevant, heating rate is subordinate within the studied experimental range. Increased heating rate from 10 to 20 °C min^−1^ increased the liquid fraction at 400 °C, likely because faster heating reduces the exposure of intermediate vapors to the reactive zone and limits the number of secondary reactions that form solid residues. This is indicated by a moderate negative correlation between heating rate and the solid products (r = −0.532) and a weak relationship with gas formation (r = −0.182). Thus, the heating rate is primarily fine-tuned under such conditions, whereas product selectivity is strongly mediated by temperature [8,11].

Overall, the yield and correlation results indicate that final temperature was the main operating variable controlling the transformation of post-consumer PS into condensable liquid products within the evaluated range. Increasing the temperature from 400 to 500 °C favored liquid recovery and reduced the relative contribution of solid and estimated gaseous fractions. This trend is supported by the correlation matrix, in which pyrolysis temperature and heating rate were nearly independent (r ≈ 0), as expected from the experimental design, in which both variables were varied independently. This separation indicates that the increase in liquid yield was mainly due to temperature, rather than to an ambiguous correlation among operating variables. The weak inverse correlation between temperature and solid products (r = −0.295) suggests that char formation decreased only slightly with increasing temperature. In contrast, the liquid and estimated gaseous fractions showed a more pronounced opposite behavior. Heating rate had a secondary effect, particularly at lower temperatures, where increasing the ramp from 10 to 20 °C min^−1^ improved liquid production. Therefore, the yield analysis identifies 500 °C and a minimum rate of 20 °C min^−1^ as the most favorable conditions for liquid recovery in this system. In contrast, the chemical composition of this liquid fraction is discussed separately in the GC–MS section [6,15,44].

Compared with catalytic and thermo-catalytic PS pyrolysis systems, the non-catalytic fixed-bed process evaluated in this study offers a simpler operational configuration because it does not require catalyst preparation, regeneration, or separation from the reaction products. Catalytic systems are widely reported to improve product selectivity, promote the formation of lighter aromatics, and, in some cases, reduce the thermal severity required for PS conversion. However, their performance may be affected by catalyst deactivation, coke formation, additional material costs, and sensitivity to impurities present in post-consumer plastic waste [7,11]. Therefore, the present non-catalytic approach should be interpreted as a baseline thermal conversion route that allows the intrinsic effects of final temperature and heating rate on PS pyrolysis to be evaluated before introducing catalytic complexity.

It should be noted, however, that the present study was not designed as a full multivariable optimization of PS pyrolysis. Parameters such as vapor residence time, N_2_ flow rate, particle-size distribution, reactor loading, and holding time can influence secondary cracking, vapor-phase reactions, condensation efficiency, and final product distribution. In this work, these variables were maintained constant to isolate the effects of final temperature and heating rate. Therefore, the best-performing condition identified here should be understood as the most favorable condition within the evaluated experimental window, rather than as a global optimum for PS pyrolysis. Future studies should include a broader experimental matrix incorporating residence time, carrier-gas flow rate, particle size, holding time, and catalytic upgrading to determine their individual and interactive effects on liquid yield and aromatic selectivity.

### 3.3. Analysis of Variance (ANOVA)

The ANOVA results, summarized in Table 5, statistically support the trends observed in the product-yield analysis. The overall model was highly significant (F = 32.90, *p* < 0.0001), indicating that the selected operating variables explained a significant fraction of the variability in liquid yield. Final temperature was the dominant factor, with a highly significant effect (F = 44.34, *p* < 0.0001) and the largest contribution to the model variability (SS = 225.40, approximately 83.0%). This result is consistent with the thermally driven depolymerization of PS, in which increasing temperature promotes main-chain C–C bond cleavage and volatilization of condensable aromatic products [15,45]. Heating rate was also statistically significant (F = 10.20, *p* = 0.0129), although its contribution was smaller (SS = 25.81, approximately 9.5%), confirming that it acted as a secondary operational parameter within the evaluated range. The model showed a high explanatory capacity, with R^2^ = 0.925, indicating that the selected factors explained 92.5% of the variation in liquid yield. In contrast, the remaining variation was attributable to residual error or uncontrolled experimental effects. Therefore, the ANOVA results provide statistical support for the yield behavior discussed above. In contrast, the compositional changes in the liquid phase are addressed separately in the GC–MS section [6,8,11,44].

### 3.4. GC-MS Analysis

The carbon-number distribution of the pyrolysis oil as a whole is shown in Table 6. This shows that the C_6_–C_11_ fraction increases with final temperature, reaching a maximum at 500 °C for both heating rates. At elevated temperatures, more light aromatic hydrocarbons are formed, and the heavier components are broken down into smaller compounds, the experiment suggests. For chemical recycling, this means that raising the final temperature increases the valuable light aromatic products in the oil phase [8,40,45,46,47]. For heavier fractions, especially C_29_–C_40_ (see Table 6), cracking of oligomeric species at higher temperatures was associated with reduced wax, which can beneficially affect downstream purification and the operating performance of the method [6,8,48]. According to Table 6, thermal intensity controls the molecular distribution of pyrolysis oil by redistributing the product spectrum from heavier oligomeric fractions to lighter aromatic compounds, thereby improving the quality of the liquid fraction for valorization [6,8,48].

**Figure 6:**
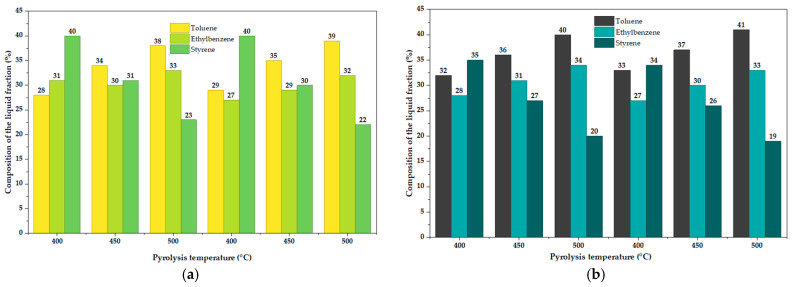
Distribution and relative composition of the main monoaromatic compounds in the PS pyrolysis oil at different heating rates: (**a**) 10 °C min^−1^ and (**b**) 20 °C min^−1^. Bar colors identify toluene, ethylbenzene, and styrene. The relative composition of these compounds is shown for each operating condition.

To provide chemical meaning to the carbon-number distribution shown in Table 6, a literature-supported assignment of probable compounds within each interval was established. This complementary analysis indicates that the C_6_–C_11_ region is mainly associated with monoaromatic species, particularly styrene, toluene, and ethylbenzene, in agreement with the compositional resolution shown in Figure 6. From a mechanistic viewpoint, this assignment is consistent with the lower kinetic demand of end-chain β-scission, which favors styrene formation, as well as with competitive hydrogen-transfer and disproportionation reactions that generate toluene and other light alkyl aromatics. In contrast, the C_12_–C_20_ interval is more reasonably attributed to dimeric aromatic products, especially 2,4-diphenyl-1-butene, whose formation requires kinetically more demanding 1,3-hydrogen transfer steps. The C_21_–C_28_ fraction can be linked to trimeric products such as 2,4,6-triphenyl-1-hexene, whereas the C_29_–C_40_ region should be interpreted more conservatively as a heavy unresolved oligomeric fraction rather than a set of directly identified molecules. Under this framework, the increase in the C_6_–C_11_ fraction with temperature, together with the decrease in the heavier intervals, supports a progressive cracking of oligomeric species and a redistribution toward lighter monoaromatic products as thermal severity increases [49,50,51].

The kinetic and energetic values summarized in Table 7 were not obtained from original calculations performed in the present study. These values were extracted from previously published experimental and theoretical studies and are used here only as literature-based support for assigning the most probable compounds and mechanistic pathways associated with each carbon-number interval. To improve traceability, the corresponding references are reported directly in brackets within the table for each energetic and mechanistic consideration.

Figure 6 provides a more detailed view of the monoaromatic composition of the liquid fraction and shows how this composition changed with increasing thermal severity. At 10 °C min^−1^ (Figure 6a), styrene was the predominant monoaromatic compound at 400 °C (about 40%), but its relative abundance decreased to 30–31% at 450 °C and to 22–23% at 500 °C. In contrast, toluene increased from 28 to 29% at 400 °C to 38–39% at 500 °C, while ethylbenzene gradually increased to 32–33% at the highest temperature. A similar but more pronounced trend was observed at 20 °C min^−1^ (Figure 6b), where toluene increased from 32 to 33% at 400 °C to 40–41% at 500 °C, ethylbenzene increased from 27% to 34%, and styrene decreased from 34 to 35% to 19–20% as temperature increased.

These results indicate that increasing temperature promoted a redistribution of the monoaromatic pool from styrene toward toluene and ethylbenzene. Mechanistically, this behavior is consistent with the known depolymerization pathway of PS, in which styrene is generated as the main primary product through radical unzipping, followed by competitive secondary reactions such as hydrogen transfer, benzylic radical stabilization, and side-chain scission, which favor the formation of toluene and ethylbenzene at higher thermal severity [6,8,11,41]. Therefore, Figure 6 complements the carbon-number analysis by showing that temperature affected not only the amount of liquid recovered, as discussed in the previous sections, but also the detailed molecular distribution of the monoaromatic fraction. From a compositional standpoint, the results suggest that pyrolysis oil may be a potential source of aromatic feedstocks or aromatic-rich fuel precursors. However, its practical use would still depend on downstream separation and purification requirements [8,44,45,52].

### 3.5. Mechanistic Interpretation of Product Distribution

#### 3.5.1. Formation Pathways of Monoaromatic Products in the C_6_–C_11_ Region

The formation of the C_6_–C_11_ fraction can be rationalized by initial random scission of the PS backbone, followed by β-scission and hydrogen-transfer reactions that collectively promote the generation of light monoaromatic products. Within this reaction network, styrene is the primary product because its formation via end-chain β-scission is kinetically favored. In contrast, toluene, ethylbenzene, and α-methylstyrene arise from secondary radical transformations involving hydrogen transfer, radical stabilization, and protonation. This mechanistic interpretation is consistent with the experimental trend observed in the present study, in which the C_6_–C_11_ fraction increases with temperature. At the same time, the relative contribution of styrene decreases due to the progressive redistribution of the monoaromatic pool toward other light aromatics under more severe thermal conditions [6,8,11,51].

**Scheme 1:**
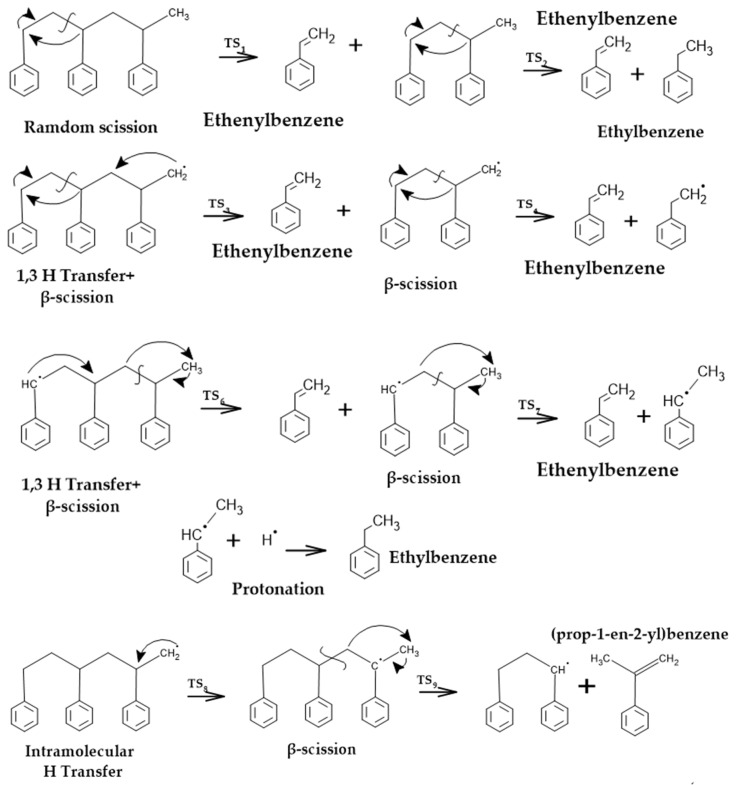
Proposed reaction pathways leading to the formation of monoaromatic products in the C_6_–C_11_ region during polystyrene pyrolysis, including styrene, ethylbenzene, toluene, and α-methylstyrene, through random chain scission, end-chain β-scission, hydrogen-transfer reactions, radical stabilization, and protonation steps.

Although styrene is one of the main target compounds in PS chemical recycling, the liquid fraction obtained in this study should not be interpreted as a purified monomer stream. As shown in Scheme 1, styrene formation is associated with the proposed monoaromatic product pathways occurring during PS pyrolysis. GC–MS analysis confirmed the presence of styrene together with other monoaromatic compounds such as toluene and ethylbenzene, as well as heavier aromatic fractions. Therefore, direct reuse of this oil for PS production would require downstream purification to isolate styrene at an appropriate purity level. In industrial practice, fractional distillation is one of the most relevant strategies for separating styrene from PS pyrolysis oil. However, additional refining steps may be required depending on the concentration of co-produced aromatics, oligomeric compounds, and impurities. No laboratory-scale separation or purification of the recovered liquid fraction was performed in the present study; therefore, the plastic-to-plastic potential is discussed as a prospective application rather than as an experimentally demonstrated closed-loop recycling route [6,8,44].

#### 3.5.2. Formation Pathways of Dimeric Products in the C_12_–C_20_ Region

**Scheme 2:**
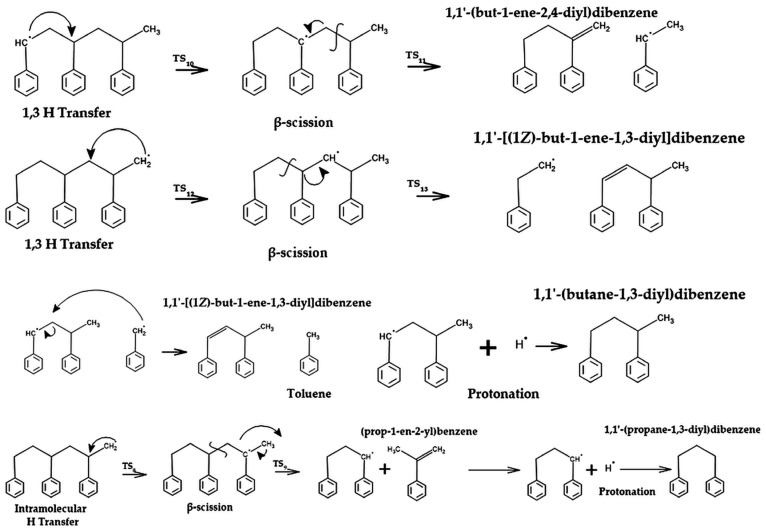
Proposed reaction pathways leading to the formation of dimeric aromatic products in the C_12_–C_20_ region during polystyrene pyrolysis, involving intramolecular hydrogen-transfer reactions, mid-chain β-scission, radical addition to olefinic chain ends, and subsequent stabilization steps, with the concurrent release of light monoaromatic coproducts.

The C12–C20 region is associated with dimeric aromatic products formed through secondary pathways that become accessible after the initial cleavage of the polymer chain, mainly through hydrogen-transfer reactions followed by mid-chain β-scission and related radical rearrangements, as shown in Scheme 2. In contrast to styrene formation, these pathways require additional structural reorganization, making dimeric products kinetically less favored than direct monomer release and explaining why they behave as competitive rather than dominant products during PS pyrolysis. This interpretation agrees with the persistence of the C12–C20 fraction in the liquid product. It supports its assignment as an intermediate mechanistic domain between monomer recovery and heavier oligomer retention, while also recognizing that alternative dominant routes for dimer formation have been proposed in previous studies [6,8,51].

## 4. Conclusions

This study assessed the thermal pyrolysis of post-consumer polystyrene in a non-catalytic batch fixed-bed reactor, focusing on the influence of final temperature and heating rate on product yield and liquid-phase composition. Feedstock characterization by TGA and micro-Raman confirmed the dominant thermal degradation behavior and chemical identity of the PS sample. In contrast, the kinetic analysis of the main TGA degradation event provided apparent activation energies of 269.18, 288.83, and 280.69 kJ mol^−1^ using the Coats–Redfern, Horowitz–Metzger, and Broido models, respectively. These values support selecting the 400–500 °C range as the relevant thermal window for PS decomposition.

Within the evaluated experimental window, final temperature was the main operating variable controlling liquid recovery. The highest liquid yield was obtained at 500 °C and 20 °C min^−1^, reaching approximately 95.2 wt%, whereas the solid fraction remained low and the gaseous fraction was estimated at approximately 2.0 wt%. The statistical analysis confirmed the dominant effect of temperature on liquid yield, while heating rate acted as a secondary but still measurable operational factor. However, because the gaseous fraction was calculated by mass balance rather than directly collected and analyzed, this value should be interpreted as an estimated gaseous fraction rather than as a fully quantified gas stream.

GC–MS analysis showed that the recovered oil was composed mainly of aromatic hydrocarbons, including styrene, toluene, and ethylbenzene. Increasing the final temperature favored the formation of lighter aromatic fractions, particularly in the C_6_–C_11_ range, while reducing the relative contribution of heavier oligomeric fractions. This behavior indicates that thermal severity not only increased liquid recovery but also promoted compositional redistribution toward lower-molecular-weight aromatic products.

The results support the technical potential of non-catalytic fixed-bed pyrolysis as a simple baseline route for producing aromatic-rich liquids from post-consumer PS. Nevertheless, the recovered oil should be considered a complex mixture rather than a purified monomer stream. Therefore, its use for styrene recovery or plastic-to-plastic recycling would require downstream separation and purification, such as fractional distillation or related refining strategies. Since no direct gas-phase characterization, life-cycle assessment, full energy balance, or techno-economic analysis was performed, the present study does not claim definitive environmental superiority or industrial circularity. Future work should address gas collection and composition, purification of the styrene-rich fraction, residence-time effects, carrier-gas optimization, catalytic upgrading, and process-scale feasibility.

## Figures and Tables

**Table 1:** Linearized equations used for the kinetic analysis of post-consumer PS degradation from TGA data.

Kinetic Model	Linearized Equation	Slope	Apparent Activation Energy
Coats–Redfern	ln[−ln(1 − α)/T^2^] vs. 1/T	m = −Ea/R	Ea = −mR/1000
Horowitz–Metzger	ln[−ln(1 − α)] vs. θ; θ = T − Ts	m = Ea/(RTs^2^)	Ea = mRTs^2^/1000
Broido	ln[ln(1/y)] vs. 1/T; y = 1 − α	m = −Ea/R	Ea = −mR/1000

**Table 2:** Apparent activation energy obtained from the kinetic models applied to the main TGA degradation region.

Kinetic Model	Slope	Ea Obtained (kJ mol^−1^)
Coats–Redfern	−32,375.040	269.18
Horowitz–Metzger	0.070430	288.83
Broido	−33,759.678	280.69

**Table 3:** Apparent activation energies obtained for each analyzed microregion and the composite TGA curve.

Curve	Ea Coats–Redfern (kJ mol^−1^)	Ea Horowitz–Metzger (kJ mol^−1^)	Ea Broido (kJ mol^−1^)
Microregion 1	297.23	311.83	309.11
Microregion 2	217.88	238.95	229.15
Microregion 3	269.18	288.83	280.69
Microregion 4	207.34	228.91	218.66
Microregion 5	285.09	303.92	296.62
Composite curve	269.18	288.83	280.69

**Table 4:** Results of the thermal pyrolysis of PS.

Polymer	Pyrolysis Temperature (°C)	Heating Rate (°C min^−1^)	Liquid Products (wt%)	Solid Products (wt%)	Estimated Gaseous Fraction (wt%)	Independent Runs
PS	400	10	80.85 ± 0.21	5.15 ± 0.21	14.05 ± 0.07	2
PS	400	20	87.25 ± 0.35	3.30 ± 0.28	9.45 ± 0.07	2
PS	450	10	87.60 ± 0.14	6.65 ± 0.21	5.75 ± 0.07	2
PS	450	20	89.00 ± 1.41	4.90 ± 1.27	6.10 ± 0.14	2
PS	500	10	94.10 ± 0.14	3.60 ± 0.14	2.30 ± 0.00	2
PS	500	20	95.10 ± 0.14	2.90 ± 0.14	2.00 ± 0.00	2

**Table 5:** Analysis of variance (ANOVA) of liquid yield in the thermal pyrolysis of PS.

	DF	Sum of Squares	Mean Square	F-Value	*p*-Value
Pyrolysis Temperature	2	225.40	112. 70	44.34	<0.0001
Heating Rate	1	25.81333	25.81	10.2	0.0129
Model	3	251.22	83.74	32.9	<0.0001
Error	8	20.33	2.54		
Total Corrected	11	271.56			

**Table 6:** Hydrocarbon distribution of pyrolysis oil for each experimental condition.

# Test	Pyrolysis Temperature (°C)	Heating Rate (°C min^−1^)	C_6_–C_11_ (%)	C_12_–C_20_ (%)	C_21_–C_28_ (%)	C_29_–C_40_ (%)	Replicate
**1**	400	10	25.0	42.0	23.0	10.0	3
**2**	400	20	26.0	41.0	23.0	10.0	3
**3**	450	10	21.0	39.0	22.0	10.0	3
**4**	450	20	22.0	38.0	22.0	10.0	3
**5**	500	10	30.0	38.0	20.0	8.0	3
**6**	500	20	31.0	37.0	20.0	8.0	3
**7**	400	10	24.0	43.0	23.0	10.0	3
**8**	400	20	27.0	40.0	23.0	10.0	3
**9**	450	10	20.0	40.0	22.0	10.0	3
**10**	450	20	23.0	37.0	22.0	10.0	3
**11**	500	10	29.0	39.0	20.0	8.0	3
**12**	500	20	32.0	36.0	20.0	8.0	3

**Table 7:** Literature-supported assignment of representative compounds across the carbon-number intervals of PS pyrolysis oil.

Carbon-Number Region	Most Probable Compounds in the Fraction	Energetic/Kinetic Support Reported in the Attached Literature	Mechanistic Justification for Assignment
C_6_–C_11_	Toluene, ethylbenzene, styrene, and α-methylstyrene are minor competitive products.	Styrene formation through end-chain β-scission has been reported as a kinetically favored route, with activation energies in the 111.2–122.0 kJ mol^−1^ range. Toluene formation has been associated with intermolecular H-transfer pathways with barriers of 42.2 and 65.9 kJ mol^−1^, while disproportionation routes are thermodynamically favorable [51].	The assignment of C_6_–C_11_ to monoaromatic products is consistent with the GC–MS identification of styrene, toluene, and ethylbenzene in the light aromatic fraction, as well as with the known predominance of styrene as a primary PS pyrolysis product [6,8,50,51].
C_12_–C_20_	2,4-diphenyl-1-butene (styrene dimer) as the principal assigned species; 1,2-diphenylcyclobutane and related dimeric aromatics as minor species	Dimer formation requires additional hydrogen-transfer and β-scission steps. Reported 1,3-H transfer barriers are in the 161.0–174.0 kJ mol^−1^ range, followed by mid-chain β-scission steps of 115.6–146.3 kJ mol^−1^ [51].	This interval is assigned to dimeric aromatic products because 2,4-diphenyl-1-butene has been experimentally reported as one of the main oligomeric primary volatiles produced during PS pyrolysis [50].
C_21_–C_28_	2,4,6-triphenyl-1-hexene (styrene trimer) as the main representative species	Styrene trimer products have been reported among the primary volatiles from PS pyrolysis, with selectivity decreasing with increasing temperature. DFT analysis also indicates that trimer cleavage toward styrene and dimer products is accompanied by high energy barriers [50,51].	The C_21_–C_28_ interval is consistent with trimeric aromatic species such as 2,4,6-triphenyl-1-hexene. Its decrease at higher temperatures supports partial cracking of heavier oligomeric products into lighter aromatic compounds [50].
C_29_–C_40_	Higher styrene oligomers and unresolved heavy aromatic species; individual molecular assignment should be conservative	Specific energetic values for individual C_29_–C_40_ species are not available in the cited mechanistic studies. Experimental literature indicates that oligomers with a degree of polymerization greater than 3 are difficult to detect by conventional GC–MS due to their high boiling points and limited elution [50].	This region should be interpreted conservatively as an unresolved heavy oligomeric/aromatic fraction rather than as a set of definitively assigned individual molecules [50].

## Data Availability

The original contributions presented in this study are included in the article. Further inquiries can be directed to the corresponding author.

## References

[1] Thakur S., Verma A., Sharma B., Chaudhary J., Tamulevicius S., Thakur V.K. (2018). Recent developments in recycling of polystyrene based plastics. Curr. Opin. Green Sustain. Chem..

[2] Schyns Z.O.G., Shaver M.P. (2020). Mechanical recycling of packaging plastics: A review. Macromol. Rapid Commun..

[3] Ragaert K., Delva L., Van Geem K. (2017). Mechanical and chemical recycling of solid plastic waste. Waste Manag..

[4] Achilias D.S. (2025). Thermo-chemical recycling of plastics as a sustainable approach to the plastic waste issue. Euro-Mediterr. J. Environ. Integr..

[5] Liu Q., Martinez-Villarreal S., Wang S., Tien N.N.T., Kammoun M., De Roover Q., Len C., Richel A. (2024). The role of plastic chemical recycling processes in a circular economy context. Chem. Eng. J..

[6] Hassibi N., Vega-Bustos Y.A., Aissaoui M.H., Mauviel G., Burklé-Vitzthum V. (2023). Thermochemical recycling of polystyrene by pyrolysis: Importance of the reflux to maximize the production of styrene and BTEX. Ind. Eng. Chem. Res..

[7] Inayat A., Fasolini A., Basile F., Fridrichova D., Lestinsky P. (2022). Chemical recycling of waste polystyrene by thermo-catalytic pyrolysis: A description for different feedstocks, catalysts and operation modes. Polym. Degrad. Stab..

[8] Zayoud A., Thi H.D., Kusenberg M., Eschenbacher A., Kresovic U., Alderweireldt N., Djokic M., Van Geem K.M. (2021). Pyrolysis of end-of-life polystyrene in a pilot-scale reactor: Maximizing styrene production. Waste Manag..

[9] Gonzalez-Aguilar M., Cabrera-Madera V.P., Vera-Rozo J.R., Riesco-Ávila J.M. (2022). Effects of heating rate and temperature on the thermal pyrolysis of expanded polystyrene post-industrial waste. Polymers.

[10] Xuan W., Yan S., Dong Y. (2023). Exploration of pyrolysis behaviors of waste plastics (polypropylene plastic/polyethylene plastic/polystyrene plastic): Macro-thermal kinetics and micro-pyrolysis mechanism. Processes.

[11] Royuela D., Martínez J.D., Callén M.S., López J.M., García T., Murillo R., Veses A. (2024). Pyrolysis of polystyrene using low-cost natural catalysts: Production and characterisation of styrene-rich pyro-oils. J. Anal. Appl. Pyrolysis.

[12] Achilias D.S., Kanellopoulou I., Megalokonomos P., Antonakou E., Lappas A.A. (2007). Chemical recycling of polystyrene by pyrolysis: Potential use of the liquid product for the reproduction of polymer. Macromol. Mater. Eng..

[13] Kumagai S., Fujiwara K., Nishiyama T., Saito Y., Yoshioka T. (2025). Chemical feedstock recovery through plastic pyrolysis: Challenges and perspectives toward a circular economy. ChemSusChem.

[14] Inayat A., Klemencova K., Grycova B., Sokolova B., Lestinsky P. (2020). Thermo-catalytic pyrolysis of polystyrene in batch and semi-batch reactors: A comparative study. Waste Manag. Res. J. A Sustain. Circ. Econ..

[15] Maafa I.M. (2021). Pyrolysis of polystyrene waste: A review. Polymers.

[16] Perez-Martinez B.B., Lopez-Urionabarrenechea A., Serras-Malillos A., Acha E., Caballero B.M., Asueta A., Arnaiz S. (2024). Influence of feedstock particle size on the certain determination of chlorine and bromine in pyrolysis oils from waste electrical and electronic equipment plastics. ACS Omega.

[17] Jiang L., Yang X.-R., Gao X., Xu Q., Das O., Sun J.-H., Kuzman M.K. (2020). Pyrolytic kinetics of polystyrene particle in nitrogen atmosphere: Particle size effects and application of distributed activation energy method. Polymers.

[18] Sánchez-Jiménez P.E., Pérez-Maqueda L.A., Perejón A., Criado J.M. (2013). Limitations of model-fitting methods for kinetic analysis: Polystyrene thermal degradation. Resour. Conserv. Recycl..

[19] Tailor R.B., Akbari V.K., Belim M.A., Desai H.N., Patel P.B., Patel P.S., Patel K.C. (2023). Study of kinetics of thermal degradation and to determine the activation energies of polyamides based on s-triazine. J. Indian Chem. Soc..

[20] Tiwari S., Gehlot C.L., Srivastava K., Srivastava D. (2021). Simulation of the thermal degradation and curing kinetics of fly ash reinforced diglycidyl ether bisphenol A composite. J. Indian Chem. Soc..

[21] Pérez-Bravo G., Contreras-Larios J.L., Rodríguez J.F., Zeifert-Soares B., Angeles-Beltrán D., López-Medina R., Vázquez-Rodríguez T., Salmones-Blasquez J. (2022). Catalytic pyrolysis process to produce styrene from waste expanded polystyrene using a semi-batch rotary reactor. Sustainability.

[22] Albor G., Mirkouei A., McDonald A.G., Struhs E., Sotoudehnia F. (2023). Fixed bed batch slow pyrolysis process for polystyrene waste recycling. Processes.

[23] Abdullah N.A., Novianti A., Hakim I.I., Putra N., Koestoer R.A. (2018). Influence of temperature on conversion of plastics waste (polystyrene) to liquid oil using pyrolysis process. IOP Conf. Ser. Earth Environ. Sci..

[24] Royuela D., Veses A., Martínez J.D., Callén M.S., López J.M., García T., Murillo R. (2024). Thermochemical recycling of polystyrene waste by pyrolysis using a pilot-scale auger reactor: Process demonstration in a relevant environment. Resour. Conserv. Recycl..

[25] Papuga S., Savković J., Djurdjevic M., Ciprioti S.V. (2024). Effect of feed mass, reactor temperature, and time on the yield of waste polypropylene pyrolysis oil produced via a fixed-bed reactor. Polymers.

[26] Zabaniotou A., Vaskalis I. (2023). Economic assessment of polypropylene waste (PP) pyrolysis in circular economy and industrial symbiosis. Energies.

[27] Djurdjevic M., Papuga S., Kolundzija A. (2023). Analysis of the thermal behavior of a fixed bed reactor during the pyrolysis process. Hem. Ind..

[28] Lee M., Ko H., Oh S. (2023). Pyrolysis of solid recovered fuel using fixed and fluidized bed reactors. Molecules.

[29] Faravelli T., Pinciroli M., Pisano F., Bozzano G., Dente M., Ranzi E. (2001). Thermal degradation of polystyrene. J. Anal. Appl. Pyrolysis.

[30] Qureshi M.S., Oasmaa A., Pihkola H., Deviatkin I., Tenhunen A., Mannila J., Minkkinen H., Pohjakallio M., Laine-Ylijoki J. (2020). Pyrolysis of plastic waste: Opportunities and challenges. J. Anal. Appl. Pyrolysis.

[31] Musivand S., Bracciale M.P., Damizia M., De Filippis P., De Caprariis B. (2023). Viable recycling of polystyrene via hydrothermal liquefaction and pyrolysis. Energies.

[32] Lee S.W., Jeong S.-J., Hidajat M.J., Kim B.-S., Hwang D.W., Yun G.-N. (2025). Sustainable chemical recycling of waste polystyrene via catalytic pyrolysis over waste shell-derived calcium oxide catalysts. ACS Omega.

[33] Luong T., Wang Y., Parmar K., Jiang C., Wang Q., Hu J. (2023). Exploring synergistic interactions between polystyrene and polyethylene. ChemPlusChem.

[34] Young R.J., Eichhorn S.J. (2008). Raman applications in synthetic and natural polymer fibers and their composites. Raman Spectroscopy for Soft Matter Applications.

[35] Meyer M.W., McKee K.J., Nguyen V.H.T., Smith E.A. (2012). Scanning angle plasmon waveguide resonance Raman spectroscopy for the analysis of thin polystyrene films. J. Phys. Chem. C.

[36] Abdelghany M. (2017). Structural and physical studies of PVC/PVDF doped nano lithium salt for electrochemical applications. J. Adv. Phys..

[37] Rodrigues L.M., Carvalho L.F.D.C.E.S., Bonnier F., Anbinder A.L., Da Silva Martinho H., Almeida J.D. (2018). Evaluation of inflammatory processes by FTIR spectroscopy. J. Med. Eng. Technol..

[38] Umurhan Y., Songsart-Power M., Limbu T.B., Phan T. (2025). Applications of Raman spectroscopy for microplastic detection and characterization: A comprehensive spectral reference. Environ. Sci. Pollut. Res..

[39] Boldrini B., Ostertag E., Rebner K., Oelkrug D. (2021). Exploring the hidden depth by confocal Raman experiments with variable objective aperture and magnification. Anal. Bioanal. Chem..

[40] De Carvalho Gomes P., Hardy M., Tagger Y., Rickard J.J.S., Mendes P., Oppenheimer P.G. (2022). Optimization of nanosubstrates toward molecularly surface-functionalized Raman spectroscopy. J. Phys. Chem. C.

[41] Fakayode S.O., Baker G.A., Bwambok D.K., Bhawawet N., Elzey B., Siraj N., Macchi S., Pollard D.A., Perez R.L., Duncan A.V. (2019). Molecular (Raman, NIR, and FTIR) spectroscopy and multivariate analysis in consumable products analysis. Appl. Spectrosc. Rev..

[42] Cebi N., Bekiroglu H., Erarslan A. (2023). Nondestructive metabolomic fingerprinting: FTIR, NIR and Raman spectroscopy in food screening. Molecules.

[43] Verma A., Pramanik H. (2024). Production of valuable chemicals via multiphase catalytic pyrolysis of hazardous waste expanded polystyrene using low cost CaCO_3_ solid base catalyst. Indian J. Chem. Technol..

[44] Yoshida E. (2023). Vacuum pyrolysis depolymerization of waste polystyrene foam into high-purity styrene using a spirit lamp flame for convenient chemical recycling. RSC Sustain..

[45] Lu C., Xiao H., Chen X. (2021). Simple pyrolysis of polystyrene into valuable chemicals. e-Polymers.

[46] Nixon K.D., Schyns Z.O.G., Luo Y., Ierapetritou M.G., Vlachos D.G., Korley L.T.J., Epps T.H. (2024). Analyses of circular solutions for advanced plastics waste recycling. Nat. Chem. Eng..

[47] Xu Z., Sun D., Xu J., Yang R., Russell J.D., Liu G. (2024). Progress and challenges in polystyrene recycling and upcycling. ChemSusChem.

[48] Liu L., Bao W., Men X., Zhang H. (2022). Engineering for life in toxicity: Key to industrializing microbial synthesis of high energy density fuels. Eng. Microbiol..

[49] Ivleva N.P., Wiesheu A.C., Niessner R. (2016). Microplastic in aquatic ecosystems. Angew. Chem. Int. Ed..

[50] Zhou J., Qiao Y., Wang W., Leng E., Huang J., Yu Y., Xu M. (2016). Formation of styrene monomer, dimer and trimer in the primary volatiles produced from polystyrene pyrolysis in a wire-mesh reactor. Fuel.

[51] Huang J., Li X., Meng H., Tong H., Cai X., Liu J. (2020). Studies on pyrolysis mechanisms of syndiotactic polystyrene using DFT method. Chem. Phys. Lett..

[52] Dhiman S., Sharma C., Kumar A., Pathak P., Purohit S.D. (2023). Microplastics in aquatic and food ecosystems: Remediation coupled with circular economy solutions to create resource from waste. Sustainability.

